# Treating invasive aspergillosis in patients with hematologic malignancy: diagnostic-driven approach versus empiric therapies

**DOI:** 10.1186/s12879-018-3584-9

**Published:** 2018-12-13

**Authors:** Rita Wilson Dib, Ray Y. Hachem, Anne-Marie Chaftari, Fady Ghaly, Ying Jiang, Issam Raad

**Affiliations:** Department of Infectious Diseases, Infection Control & Employee Health, Unit 1460, The University of Texas MD Anderson Cancer Center, 1515 Holcombe Blvd, Houston, TX USA

**Keywords:** Invasive aspergillosis, Diagnostic driven therapy, Empiric therapy, Voriconazole, Cancer

## Abstract

**Background:**

Early antifungal therapy for invasive aspergillosis (IA) has been associated with improved outcome. Traditionally, of empiric antifungal therapy has been used for clinically suspected IA. We compared outcomes of patients with hematologic malignancy and IA who were treated with voriconazole using the diagnostic driven DDA (DDA-Vori) that includes galactomannan testing vs. empiric therapy with a non-voriconazole-containing regimen (EMP-non-Vori) or empiric therapy with voriconazole (EMP-Vori).

**Methods:**

We retrospectively reviewed the medical records of 342 hematologic malignancy patients diagnosed with proven, or probable IA between July 1993 and February 2016 at our medical center who received at least 7 days of DDA-Vori, EMP-Vori, or EMP-non-Vori. Outcome assessment included response to therapy (clinical and radiographic), all-cause mortality, and IA-attributable mortality.

**Results:**

By multivariate analysis, factors predictive of a favorable response included localized/sinus IA vs. disseminated/pulmonary IA (*p* <  0.0001), not receiving white blood cell transfusion (*p* <  0.01), and DDA-Vori vs. EMP-non-Vori (*p* < 0.0001). In contrast, predictors of mortality within 6 weeks of initiating IA therapy included disseminated/pulmonary infection vs. localized/sinus IA (*p* < 0.01), not undergoing stem cell transplantation within 1 year before IA (*p* = 0.01), and EMP-non-Vori vs. DDA-Vori (*p* < 0.001).

**Conclusions:**

DDA-Vori was associated with better outcome (response and survival) compared with EMP-non-Vori and with equivalent outcome to EMP-Vori in hematologic malignancy patients. These outcomes associated with the implementation of DDA could lead to a reduction in the unnecessary costs and adverse events associated with the widespread use of empiric therapy.

## Background

Invasive aspergillosis (IA) in patients with hematologic malignancies and in patients undergoing hematopoietic stem cell transplantation (HSCT) is still associated with high morbidity and mortality rates [1–3]. Early antifungal therapy has been associated with better outcomes; however, diagnosing IA with use of conventional diagnostic methods can be challenging because of the nonspecific clinical features of the disease and because the diagnosis is histopathologically and microbiologically complicated and often unfeasible. These hurdles led to the current practice of early initiation of empiric antifungal therapy with an anti-mold agent in patients with suspected IA. However, this excessive and non-targeted therapy may lead to unnecessary adverse effects and high medical costs while increasing the risk of antifungal resistance [4, 5].

In recent years, novel diagnostic tools have been evaluated to improve the assessment and treatment of patients with IA. The sensitivity of Aspergillus galactomannan (GM) enzyme immunoassay testing is 82% (ranges from 73 to 90%) and its specificity is 81% (ranges from 72 to 90%) in the immunocompromised population who are not receiving anti-mold therapy or prophylaxis [6, 7]. In addition, specific radiological findings detected on high-resolution computed tomographic (CT) scans highly correlated with the presence of fungal infection [8, 9]. These advances in diagnostic techniques, may help the clinician to establish an early diagnosis and rapidly initiate a diagnostic driven treatment instead of the current empiric approach that may lead to unnecessary treatment to certain patients.

Voriconazole is now the preferred anti-mold agent for primary therapy of IA, particularly in high-risk patients. Recommended alternative agents include liposomal amphotericin B caspofungin and/or isavuconazole [10].

The purpose of this study was to compare the diagnostic-driven approach (DDA) using voriconazole (DDA-Vori) with the currently used empiric approach consisting of either a regimen that did not contain voriconazole (EMP-non-Vori) or one that did contain voriconazole (EMP-Vori) as primary therapy for IA diagnosed on the basis of radiographic findings and positive GM antigen test or microbiological culture.

## Methods

This was a retrospective study of patients with hematological malignancies that were diagnosed with probable or proven invasive aspergillosis at our institution from July 1993 to February 2016. This study was approved by our Institutional Review Board and waiver of informed consent was granted.

From MD Anderson’s microbiology laboratory database, we extracted all patients (*n* = 604) who had had either hematologic malignancies or were post-HSCT with evidence of Aspergillus species growing in cultures obtained from sites of infection driven by clinical and radiological clues. We included patients equal and older than 14 years of age. Proven or probable IA was classified according to criteria from the European Organization for Research and Treatment of Cancer/Invasive Fungal Infections Cooperative Group and the National Institute of Allergy and Infectious Diseases Mycoses Study Group (EORTC/MSG) [11]. Patients with other types of malignancies such as solid tumors were excluded. We collected datafrom electronic patient medical records including relevant information on HSCT performed within the year prior to the diagnosis as well as antifungal therapy received for the treatment of IA, and prophylaxis if applicable. We excluded patients who received less than 7 days of antifungal therapy.

The diagnosis was based on positive aspergillus culture before 2002 or either a positive GM result (GM considered positive if index value was above 0.8 or 2 consecutive values ranged between 0.5 and 0.8) whether from serum [12] or from bronchoalveolar lavage (BAL) [13] and/or positive fungal culture for the period after 2002 since GM test was available at our institution. Patients with pulmonary diseases should have a CT scan suggestive of invasive fungal infection. Outcome information included response to therapy, all-cause mortality, IA-attributable mortality, and adverse events.

We compared the efficacy of therapy in patients who received DDA-Vori regimen with those who received an EMP-non-Vori regimen as well as with patients who received an EMP-Vori regimen. The main antifungal in the DDA-vori and EMP-Vori regimen consisted of voriconazole whereas the main antifungal in the non-vori group consisted of polyenes, echinocandins or other azoles than voriconazole.

### Definitions

*Diagnostic-driven therapy* was defined as any therapy initiated in patients with underlying malignancy or in those who underwent HSCT provided that therapy was initiated after the diagnosis of IA was established, this approach included the ‘pre-emptive’ component defined previously by authors in similar studies [5, 14].

In *empiric therapy*, anti-mold therapy was initiated in patients before a positive GM or fungal culture was obtained ignited by clinical and radiological drivers.

*Breakthrough infection is noted* upon the occurrence of proven or probable invasive aspergillosis while the patient is on prophylaxis.

*Response to primary therapy* was defined as improvement or stabilization of the clinical signs and symptoms assessed by the treating physician and or radiological findings after receiving at least 7 days of same antifungal therapy.

### Statistical methods

Categorical variables were compared using Chi-square or Fisher’s exact test, as appropriate. Continuous variables were compared using Wilcoxon rank sum test. Logistic regression analysis was used to evaluate the independent effect of primary therapy on response. Similarly, Cox proportional hazards regression analysis and competing risk analysis were used to evaluate the independent effect of primary therapy on all-cause mortality and IA-attributable mortality, respectively. In addition, Kaplan-Meier survival curves were estimated for patients with different primary therapies and compared using a log-rank test. The cumulative incidence curves of IA-attributable mortality were also estimated with use of competing risk analysis. All tests were two-sided with a significance level of 0.05. Statistical analyses were performed using SAS version 9.3 (SAS Institute Inc., Cary, NC) and R version 2.15. 0 (R Development Core Team, 2008).

## Results

A total of 342 patients fulfilled all inclusion criteria and were included in the study. They consisted of 44 in the DDA-Vori group, 221 in the EMP-non-Vori group, and 77 in the EMP-Vori group. Patients’ clinical characteristics, response to treatment, all-cause and IA-associated mortalities were analyzed.

### Basic characteristics of the three groups

The majority of IA cases had invasive pulmonary infection (84%) and fulfilled the criteria for proven or probable disease. Overall, 75% were patients with leukemia and 39% had undergone HSCT. *Aspergillus fumigatus* was the most common isolate observed in the patients (36%), followed by *Aspergillus terreus* (22%), *Aspergillus flavus* (21%), and *Aspergillus niger* (13%). The fungal species isolated also included *Aspergillus versicolor* and *Aspergillus nidulans*. The three groups were similar with respect to gender and IA categories.

Patients in both empiric groups were more likely to have neutropenia at the time of infection compared with the diagnostic-driven group (56% for EMP-non-Vori vs 19% for DDA-Vori, *p* < 0.0001; 40% for EMP-Vori vs 19% for DDA-Vori, *p* = 0.02). It is to note that 80.5% of the patients in the EMP-non-Vori group received an antifungal regimen that contained the lipid formulation of amphotericin B. In addition, the two empiric groups significantly received more active immunotherapy when compared with the DDA group (70% for EMP-non-Vori vs 23% for DDA-Vori, *p* < 0.0001; 49% for EMP-Vori vs 23% for DDA-Vori, *p =* 0.006). They were also more likely to have received prophylactic antifungal treatment before therapy (35% for EMP-non-Vori vs 16% for DDA-Vori, *p* = 0.012, 36% for EMP-Vori vs 16% for DDA-Vori, *p* = 0.022).

By univariate analysis, the response to therapy was significantly higher in the DDA-Vori group (73%) than in the EMP-Vori (51%, *p* = 0.018) or EMP-non-Vori (14%, *p* < 0.0001) groups. All-cause mortality was not different between the two voriconazole-based therapies (5% vs 9%, *p* = 0.48) but was significantly different when DDA-Vori therapy was compared with EMP-non-Vori therapy (5% vs 56%, *p* < 0.0001) (Tables 1 and 2). Similar results found by analyzing IA-attributable mortality.

**Table 1:** Comparing patients under diagnostic-driven therapy with voriconazole and those under empiric antifungal therapy without voriconazole

Characteristics and outcomes	Diagnostic-driven therapy with voriconazole	Empiric antifungal therapy without voriconazole	*p*-value
	(*n* = 44)	(*n* = 221)	
	*N* (%)	*N* (%)	
Age (years), median (range)	63 (23–81)	51 (14–80)	< 0.001
Gender, male	26 (59)	131 (59)	0.98
Diagnosis of IA			< .001
Definite IA	4/43 (9)	83 (38)	
Probable IA	39/43 (91)	138 (62)	
Invasive pulmonary infection^a^	40 (91)	178 (81)	0.10
Disseminated infection^a^	2 (5)	18 (8)	0.41
Localized or sinus infection^a^	4 (9)	28 (13)	0.51
Leukemia	19 (43)	181/220 (82)	< .0001
Lymphoma	16 (36)	34/220 (15)	0.001
Myeloma	8 (18)	5/220 (2)	< .001
Transplantation within 1 year prior to infection	16 (36)	82/220 (37)	0.91
Type of transplantation within prior year			0.010
Allogeneic transplant	10/16 (63)	74/82 (90)	
Autologous transplant	6/16 (38)	8/82 (10)	
Graft vs Host Disease (GVHD)	8/10 (80)	52/74 (70)	0.72
Neutropenia (< 500 ANC) at onset of IA	8/42 (19)	120/216 (56)	< .0001
Persistent neutropenia	14/36 (39)	87/210 (41)	0.77
Received immunotherapy	10/43 (23)	154/220 (70)	< .0001
Received WBC transfusion	2/42 (5)	45 (20)	0.016
Year of IA diagnosis/treatment			< .0001
1993–2004	8 (18)	162 (73)	
2005–2016	36 (82)	59 (27)	
Prophylactic antifungal treatment prior to	7 (16)	78 (35)	0.012
infection			
Breakthrough	6/7 (86)	67/78 (86)	> .99
Response to therapy	32 (73)	30 (14)	< .0001
Death within 42 days of starting therapy	2 (5)	123/220 (56)	< .0001
Aspergillosis-attributable death within 42 days of starting therapy	2 (5)	107/218 (49)	< .0001

^a^One patient had all 3 types of IA infections and 3 patients had both invasive pulmonary and localized or sinus infections

**Table 2:** Comparing patients under diagnostic-driven therapy with voriconazole and those under empiric antifungal therapy with voriconazole Characteristics

Characteristics and outcomes	Diagnostic-driven therapy with voriconazole	Empiric antifungal therapy with voriconazole	*p* - value
	(*n* = 44)	(*n* = 77)	
	*N* (%)	*N* (%)	
Age (years), median (range)	63 (23–81)	58 (22–86)	0.10
Gender, male	26 (59)	46 (60)	0.94
Diagnosis of IA			0.06
Definite IA	4/43 (9)	18 (23)	
Probable IA	39/43 (91)	59 (77)	
Invasive pulmonary infection ^a^	40 (91)	68 (88)	0.77
Disseminated infection^a^	2 (5)	6 (8)	0.71
Localized or sinus infection^a^	4 (9)	3 (4)	0.25
Leukemia	19 (43)	55 (71)	0.002
Lymphoma	16 (36)	16 (21)	0.06
Myeloma	8 (18)	4 (5)	0.029
Transplantation within 1 year prior to infection	16 (36)	34 (44)	0.40
Type of transplantation within prior year			0.14
Allogeneic transplant	10/16 (63)	29/34 (85)	
Autologous transplant	6/16 (38)	5/34 (15)	
Graft vs Host Disease (GVHD)	8/10 (80)	22/29 (76)	> .99
Neutropenia (< 500 ANC) at onset of IA	8/42 (19)	29/73 (40)	0.022
Persistent neutropenia	14/36 (39)	21/64 (33)	0.54
Received immunotherapy	10/43 (23)	37/76 (49)	0.006
Received WBC transfusion	2/42 (5)	8 (10)	0.49
Year of IA diagnosis/treatment			0.73
1993–2004	8 (18)	16 (21)	
2005–2016	36 (82)	61 (79)	
Prophylactic antifungal treatment prior to	7 (16)	27/76 (36)	0.022
infection			
Breakthrough	6/7 (86)	25/26 (96)	0.38
Response to therapy	32 (73)	39 (51)	0.018
Death within 42 days of starting therapy	2 (5)	7/76 (9)	0.48
Aspergillosis-attributable death within 42 days of starting therapy	2 (5)	6/76 (8)	0.71

^a^One patient had all 3 types of IA infections

### Predictors of response to primary therapy

By multivariate analysis, factors independently associated with response to therapy included the type of IA infection (*p* < 0.001), whereby pulmonary and disseminated infections were 0.12 (95% confidence interval [CI]: 0.04 to 0.34) and 0.02 (95% CI: < 0.01 to 0.16) times as likely to have a response when compared with localized/sinus infection, respectively (Table 3).

**Table 3:** Predictors of response to primary therapy in patients with hematologic malignancy and aspergillosis

Variables	Univariate analysis			Multivariate analysis	
	Response (*n* = 101) *N* (%)	No-Response (*n* = 241) *N* (%)	*p*-value	OR (95% CI)	*p*-value
Age (years), median (range)	59 (14–81)	52 (16–86)	0.007		–
Gender, male	60 (59)	143 (59)	0.99		
Diagnosis of invasive aspergillosis			0.024		–
Definite	22/100 (22)	83 (34)			
Probable	78/100 (78)	158 (66)			
Type of IA infection ^a^			< .001		< .0001
Invasive pulmonary infection	84 (83)	202 (84)		0.12 (0.04, 0.34)	(< .0001)
Disseminated infection	1 (1)	24 (10)		0.02 (< 0.01, 0.16)	(< .0001)
Localized or sinus infection	16 (16)	15 (6)		Referent	
Type of cancer			0.03		–
Leukemia	66/98 (67)	189/240 (79)			
Lymphoma	23/98 (23)	43/240 (18)			
Myeloma	9/98 (9)	8/240 (3)			
Transplantation within 1 year prior to infection	34 (34)	98/240 (41)	0.21		–
Type of transplantation within prior year			0.26		
Allogeneic transplant	27/34 (79)	86/98 (88)			
Autologous transplant	7/34 (21)	12/98 (12)			
Graft vs Host Disease (GVHD)	18/27 (67)	64/86 (74)	0.43		–
Neutropenia (< 500 ANC) at onset of IA	31/94 (33)	126/237 (53)	0.001		–
Persistent neutropenia	30/79 (38)	92/231 (40)	0.77		
Received immunotherapy	38/99 (38)	163/240 (68)	< .0001		–
Received WBC transfusion	5/100 (5)	50/240 (21)	< .001	0.21 (0.05, 0.66)	0.005
Year of IA diagnosis/treatment			< .0001		–
1993–2004	32 (32)	154 (64)			
2005–2016	69 (68)	87 (36)			
Prophylactic antifungal treatment prior to infection	23/100 (23)	89 (37)	0.013		–
Breakthrough	22/23 (96)	76/88 (86)	0.30		
Primary therapy strategy			< .0001		< .0001
Diagnostic-driven therapy with voriconazole	32 (32)	12 (5)		Referent	
Empiric antifungal therapy without voriconazole	30 (30)	191 (79)		0.05 (0.02, 0.12)	(< .0001)
Empiric antifungal therapy with voriconazole	39 (39)	38 (16)		0.41 (0.16, 1.02)	(0.06)

^a^All patients with more than one types of IA infections in this study had invasive pulmonary infection and were included in invasive pulmonary infection category in this analysis

Patients receiving white blood cells were less likely to have a favorable response than were those not receiving white blood cells (OR = 0.21, 95% CI: 0.05 to 0.66, *p* = 0.005).

Multivariate analysis also showed a significant effect of primary therapy on response (*p* < .0001). Patients in the EMP-non-Vori group were less likely to have a favorable response compared with those in the DDA-Vori group (OR = 0.05, 95% CI: 0.02 to 0.12, *p* < 0.0001). In contrast, there was no significant difference in response between the two Vori groups (*p* = 0.06) (Table 3).

### Predictors of mortality within 6 weeks of initiation of IA therapy

A definite diagnosis was a significant predictor of all-cause mortality at 6 weeks (HR 1.5, 95% CI: 1.1 to 2.2). Invasive pulmonary disease and disseminated disease were 3.3 (95% CI: 1.5 to 7.2) and 4.0 (95% CI: 1.6 to 10.3) times more likely to be associated with overall mortality than localized or sinus infection respectively. Having undergone HSCT within 1 year of IA infection was associated with a lower mortality rate (HR 0.60, 95% CI: 0.41 to 0.88, *p* = 0.008). Patients with anti-mold antifungal prophylaxis were also less likely to have an all-cause mortality (HR = 0.61, 95% CI: 0.41 to 0.90). Patients treated with EMP-non-Vori were 18.8 times (95% CI: 4.4 to 72.4, *p* < 0.0001) more likely to die than were those in the DDA-Vori group. Empiric and diagnostic-driven therapies with voriconazole were comparable in mortality (*p* = 0.34) (Table 4).

**Table 4:** Predictors of mortality within 42 days of initiation of IA therapy in patients with hematologic malignancy and aspergillosis

Variables	Univariate			Multivariate analysis	
	Dead (*n* = 132) *N* (%)	Alive (*n* = 208) *N* (%)	*p*-value	HR (95% CI)	*p*-value
Age (years), median (range)	50 (16–80)	56 (14–86)	0.07		–
Gender, male	84 (64)	119 (57)	0.24		–
Diagnosis of invasive aspergillosis			0.001		0.023
Definite	54 (41)	50/207 (24)		1.5 (1.1, 2.2)	
Probable	78 (59)	157/207 (76)		Referent	
Type of IA infection ^a^			0.16		0.007
Invasive pulmonary infection	111 (84)	174 (84)		3.3 (1.5, 7.2)	(0.003)
Disseminated infection	13 (10)	12 (6)		4.0 (1.6, 10.3)	(0.003)
Localized or sinus infection	8 (6)	22 (11)		Referent	
Type of cancer			0.05		–
Leukemia	106/131 (81)	148/205 (72)			
Lymphoma	23/131 (18)	43/205 (21)			
Myeloma	2/131 (2)	14/205 (7)			
Transplantation within 1 year prior to infection	41/131 (31)	91 (44)	0.022	0.60 (0.41, 0.88)	0.008
Type of transplantation within prior year			0.06		
Allogeneic transplant	39/41 (95)	74/91 (81)			
Autologous transplant	2/41 (8)	17/91 (19)			
Graft vs Host Disease (GVHD)	29/39 (74)	53/74 (72)	0.76		
Neutropenia (< 500 ANC) at onset of IA	79/130 (61)	76/199 (38)	< .0001		–
Persistent neutropenia	59/130 (45)	63/179 (35)	0.07		–
Received immunotherapy	92 (70)	108/205 (53)	0.002		–
Received WBC transfusion	31 (23)	24/206 (12)	0.004		–
Year of IA diagnosis/treatment			< .0001		–
1993–2004	99 (75)	87 (42)			
2005–2016	33 (25)	121 (58)			
Prophylactic antifungal treatment prior to infection	36 (27)	76/207 (37)	0.07	0.61 (0.41, 0.90)	0.012
Breakthrough	29/36 (81)	69/75 (92)	0.11		
Primary therapy strategy			< .0001		< .0001
Diagnostic-driven therapy with voriconazole	2 (2)	42 (20)		Referent	
Empiric antifungal therapy without voriconazole	123 (93)	97 (47)		18.0 (4.4, 73.2)	(< .0001)
Empiric antifungal therapy with voriconazole	7 (5)	69 (33)		2.2 (0.5, 10.6)	(0.33)

*IA* = Invasive aspergillosis; *WBC* = White blood cell; *HR* = Hazard ratio; 95% *CI* = 95% Confidence interval

^a^All patients with more than one types of IA infections in this study had invasive pulmonary infection and were included in invasive pulmonary infection category in this analysis

Regarding IA-attributed mortality, we found similar independent predictors. In addition, having had the infection during the years 1993 to 2004 was associated with a 1.8 times (95% CI: 1.1 to 2.8) higher likelihood of IA-attributable mortality than was having had the infection in later years (2005–2016) (*p* = 0.012). Compared with the DDA-Vori group, patients receiving EMP-non-Vori were 9.8 times (95% CI: 2.4 to 40.7) more likely to die of the infection (*p* = 0.002), and those receiving EMP-Vori showed no significant difference (*p* = 0.51).

**Fig. 1:**
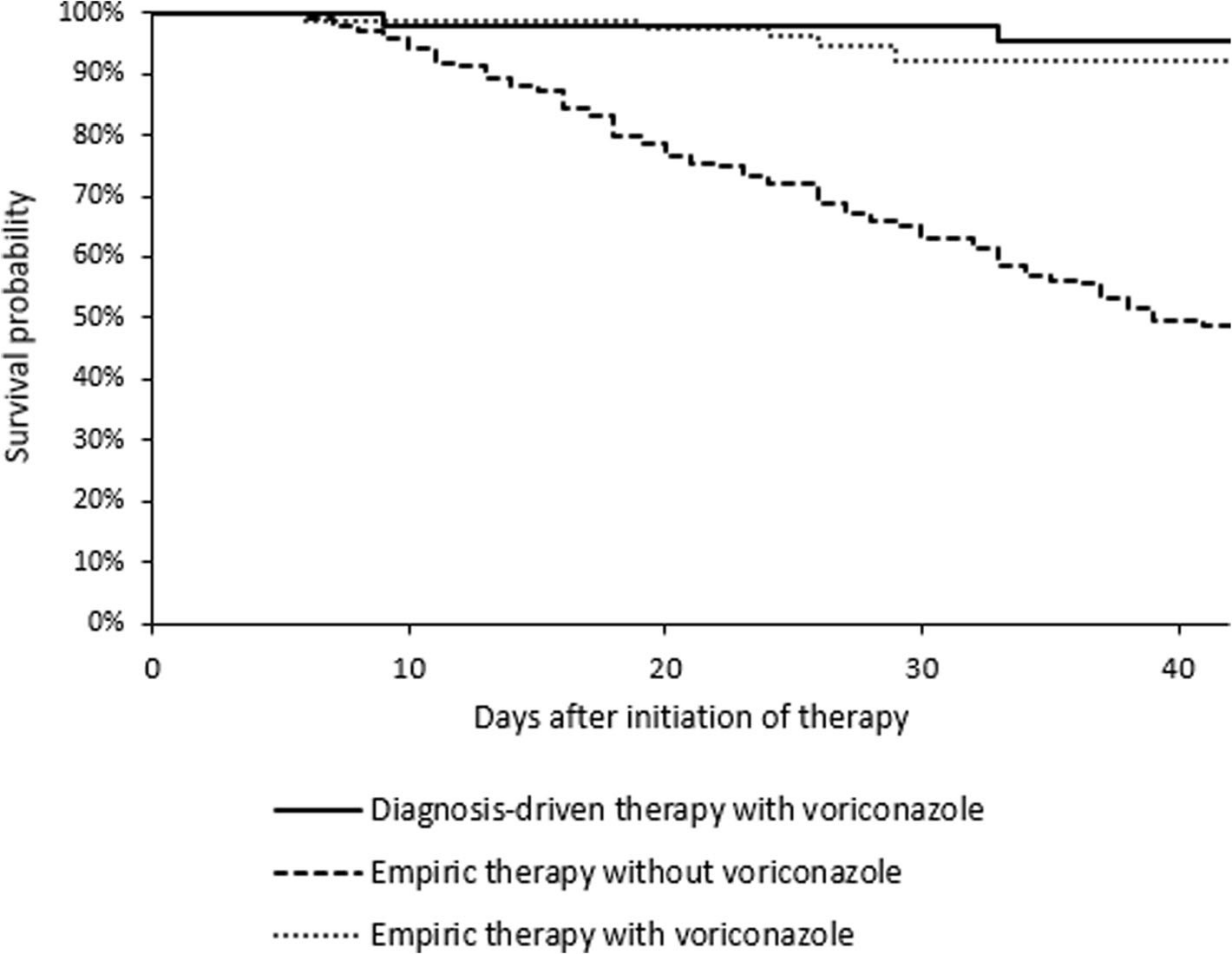
Kaplan-Meier Survival Curves of Patients with Different Therapies

Kaplan-Meier survival analysis also showed that the DDA-Vori group was associated with better survival rates than was the EMP-non-Vori group (*p* < 0.0001), but there was no significant difference between the DDA-Vori group and the EMP-Vori group (*p* = 0.47) (Fig. 1).

The duration (days) between IA diagnosis and starting primary therapy for the DDA-Vori group showed an interquartile range of 6 to 19 days and a median of 8 days. One hundred and nine patients in the study received GM tests within +/− 1 week of IA diagnosis, of whom 30 had positive results and 79 had negative results. Patients with positive GM tests had numerically but not statistically worse outcomes than those with negative GM tests, including response to primary therapy (33% vs 44%, *p* = 0.30), all-cause mortality (34% vs 21%, *p* = 0.13) and IA-attributed mortality (21% vs 17%, *p =* 0.59).

## Discussion

In our study, we found that DDA-Vori therapy for IA based on GM biomarkers and microbiologic evidence had significantly better survival rates and response rates than did the non-vori–containing empiric approach. In addition, the DDA-Vori approach had equivalent outcomes (response and survival) to those observed with EMP-Vori therapy. It is also important to note that the median 8-day time delay between diagnosis and treatment in the DDA-Vori treatment group did not impact negatively on the outcomes compared to immediate empirical anti-fungal therapy.

The first randomized trial to support empiric antifungal therapy was published in 1982 [15]. To this day, the empiric approach is accepted as the standard of care in most daily clinical practices [16]. Several attempts were made to base antifungal therapy on a diagnostic strategy [14, 17–21]. In 2005, Maertens et al. [19] performed a diagnostic evaluation encompassing clinical, serial GM, and microbiological factors in a high-risk population with neutropenia and fever. Maertens followed a protocol whereby only those who had clinical signs and symptoms and tested positive were treated with liposomal amphotericin B. This resulted in a decrease in the treatment rate, from 35 to 7%, with no reported missed cases of IA. However, one case of zygomycosis was missed and diagnosed post-mortem. In addition, the study was conducted in a small subgroup of patients [19].

Girmenia et al. [22] designed an intensive diagnostic workup strategy that led to 43% decreased use of antifungal therapy with no undetected invasive fungal disease at 3 months after follow-up in 159 cases of neutropenic fever. In contrast, Cordonnier et al. [14] showed that overall mortality rate in the group treated with amphotericin B according to a preemptive or diagnostic strategy based on clinical factors or GM in persistent neutropenia was not inferior to that in the group treated empirically.

Another randomized trial done in multiple Australian centers showed that the use of biomarkers to treat suspected IA has decreased the rates of empiric therapy without affecting mortality rates [20]. Aguilar-Guisado et al. [17] conducted a study in hematologic malignancy patients with persistent febrile neutropenia and administered antifungal therapy only to the group who fulfilled a set of diagnostic criteria. In that study, 38% of the patients were not treated with antifungal drugs, and none in this subgroup had a final diagnosis of a fungal infection. However, in the treated group, 40% had non-fungal infections and 42.3% had a diagnosis of probable or possible IA; their primary therapy consisted mainly of caspofungin and voriconazole based on the most likely fungal etiology [17].

Consistent with findings in previous reports [23–25], we also demonstrated that DDA-Vori therapy was associated with better outcome in terms of response and IA-attributable mortality when compared with EMP-non-Vori. As noted earlier, most (80.5%) of the patients who received EMP-non-Vori were on a regimen that included a lipid formulation of amphotericin B. DDA-Vori therapy was associated with equivalent outcome when compared with EMP-Vori therapy.

Voriconazole has been suggested as the primary treatment for invasive pulmonary and extrapulmonary aspergillosis by the Infectious Disease Society of America [10]. When voriconazole treatment was compared with amphotericin B as primary treatment for possible, probable, and proven IA infections, it showed better survival and response rates among the proven and probable infection groups and showed better response rates in the possible infection group [26]. The fact that EMP-Vori and DDA-Vori were not statistically different further emphasizes the importance of targeted therapy using the appropriate agent and its role in preventing overtreatment without compromising care in this critical patient population. We also noticed that the decreased mortality observed from the year 2004 corresponded to the period when voriconazole was introduced in our medical center.

Anti-mold prophylaxis was found to be a positive predictor of survival. It is possible that anti-mold prophylaxis partially decreases the fungal burden in infected patients, hence ameliorating the course of IA. This is evident by the fact that the aspergillus galactomannan enzyme immunoassay is markedly affected by concomitant administration of prophylactic anti-mold drugs. It is a well-known fact that GM levels are directly proportional to the degree of fungal burden and hence prognostically predict a worst outcome [4, 7, 27].

The excessive use of antifungals, however, in the empiric treatment of suspected possible IA could play a role in the emergence of resistance (particularly azole resistance) among *Aspergillus* species, which is associated with treatment failure and elevated rates of breakthrough infections that are difficult to treat with an azole-containing regimen [28]. Multiple point mutations have been determined at the molecular level among strains recovered from patients receiving triazole anti-mold treatment. The resulting amino acid substitutions are believed to be associated with the emerging *Aspergillus* antifungal resistance [29].

In addition, there could be a cost benefit associated with the DDA. A meta-analysis published in 2015 assessed and compared the total costs associated with empiric and preemptive therapy, in which most of the included studies were based on GM and imaging studies. This analysis found an average reduction of $324 per patient of the total cost, considering the workup and antifungal medications [30]. This needs to be further evaluated in future prospective studies at our center.

This study had some limitations because of its retrospective nature and because it was conducted in a single medical center. Compared to the other two groups, the sample size of DDA-Vori group was small (*n* = 44). We didn’t account for the status of the malignant disease at the time of diagnosis (remission vs relapse/refractory disease), intensity of Graft vs Host Disease (GVHD) and comorbidities/coinfections.

The strength of this study was its inclusion of a large cohort of hematologic malignancy patients and post-HSCT patients having probable or proven IA. However, the numbers of patients receiving DDA-Vori was smaller than the numbers receiving the other two therapies, which was another limitation. The results apply to our population of hematological malignancies and HSCT but cannot be extrapolated to solid organ transplant or other populations.

## Conclusions

In conclusion, the DDA could reduce the use of unnecessary antifungals with a superior to equivalent outcome. This approach allows for a more rational utilization of available antifungals with potential reduction in costs and adverse events associated with the widespread use of empiric therapy. In addition, this targeted diagnostic-driven therapy could lead to improved quality of care in this high-risk patient population.

## Abbreviations

BAL: Bronchoalveolar Lavage
CT: Computed Tomographic
DDA: Diagnostic-Driven Approach
DDA-Vori: Diagnostic Driven DDA
EMP-Non-Vori: Non-Voriconazole-Containing Regimen
EMP-Vori: Empiric Therapy With Voriconazole
EORTC/MSG: European Organization For Research And Treatment Of Cancer/Invasive Fungal Infections Cooperative Group And The National Institute Of Allergy And Infectious Diseases Mycoses Study Group
GM: Galactomannan
HSCT: Hematopoietic Stem Cell Transplantation
IA: Invasive Aspergillosis
